# Survival and Estimation of Direct Medical Costs of Hospitalized COVID-19 Patients in the Kingdom of Saudi Arabia (Short Title: COVID-19 Survival and Cost in Saudi Arabia)

**DOI:** 10.3390/ijerph17207458

**Published:** 2020-10-13

**Authors:** Anas A. Khan, Yazed AlRuthia, Bander Balkhi, Sultan M. Alghadeer, Mohamad-Hani Temsah, Saqer M. Althunayyan, Yousef M. Alsofayan

**Affiliations:** 1 Department of Emergency Medicine, College of Medicine, King Saud University, Global Center for Mass Gatherings Medicine, Ministry of Health, Riyadh 11451, Saudi Arabia; anaskhan@KSU.EDU.SA; 2 Department of Clinical Pharmacy, College of Pharmacy, King Saud University, P.O. Box 2454, Riyadh 11451, Saudi Arabia; bbalkhi@KSU.EDU.SA (B.B.); salghadeer@ksu.edu.sa (S.M.A.); 3 Pharmacoeconomics Research Unit, College of Pharmacy, King Saud University, P.O. Box 2454, Riyadh 11451, Saudi Arabia; 4 College of Medicine, King Saud University, Riyadh 11451, Saudi Arabia; mtemsah@ksu.edu.sa; 5 Department of Pediatrics, King Saud University Medical City, King Saud University, Riyadh 11451, Saudi Arabia; 6 Department of Accident and Trauma, Prince Sultan Bin Abdulaziz College for Emergency Medical Services, King Saud University, Riyadh 11451, Saudi Arabia; salthunayyan@KSU.EDU.SA; 7 Global Center for Mass Gatherings Medicine, Ministry of Health, Riyadh 11451, Saudi Arabia; y-m-alsofayan@hotmail.com

**Keywords:** COVID-19, hospitalization, mortality, health care costs, Saudi Arabia

## Abstract

Objectives: Assess the survival of hospitalized coronavirus disease 2019 (COVID-19) patients across age groups, sex, use of mechanical ventilators (MVs), nationality, and intensive care unit (ICU) admission in the Kingdom of Saudi Arabia. Methods: Data were retrieved from the Saudi Ministry of Health (MoH) between 1 March and 29 May 2020. Kaplan–Meier (KM) analyses and multiple Cox proportional-hazards regression were conducted to assess the survival of hospitalized COVID-19 patients from hospital admission to discharge (censored) or death. Micro-costing was used to estimate the direct medical costs associated with hospitalization per patient. Results: The number of included patients with complete status (discharge or death) was 1422. The overall 14-day survival was 0.699 (95%CI: 0.652–0.741). Older adults (>70 years) (HR = 5.00, 95%CI = 2.83–8.91), patients on MVs (5.39, 3.83–7.64), non-Saudi patients (1.37, 1.01–1.89), and ICU admission (2.09, 1.49–2.93) were associated with a high risk of mortality. The mean cost per patient (in SAR) for those admitted to the general Medical Ward (GMW) and ICU was 42,704.49 ± 29,811.25 and 79,418.30 ± 55,647.69, respectively. Conclusion: The high hospitalization costs for COVID-19 patients represents is a significant public health challenge. Efficient allocation of healthcare resources cannot be emphasized enough.

## 1. Introduction

Coronavirus disease 2019 (COVID-19) has affected every continent on Earth, and the number of confirmed cases has exceeded 9 million worldwide [1]. As more details about COVID-19 and its associated risk factors have surfaced, the diagnostic and clinical features, treatment, typical clinical course, and monitoring which distinguish the virus that causes COVID-19, severe acute respiratory syndrome coronavirus 2 (SARS-CoV-2), have become clear. However, there remain inconsistencies in disease severity among patients and mortality among different countries that hamper the assessment and triage of patients [2,3,4,5,6]. 

The overall case fatality rate (CFR) of COVID-19 has been estimated be ~0.7% (95% confidence interval (CI): 0.4–1.0) and to range from 0.001% to 10.1% among those under 20 and over 80 years of age, respectively [7]. Moreover, it has been reported that the CFR can reach as high as 17% in the Northern regions of Italy [8]. In China, Yan-ni and colleagues estimated the CFR to be 6.1 ± 2.9% [9]. However, the CFR can be as high as 28% among hospitalized patients [10]. 

Although the rate of hospitalization among patients confirmed to have COVID-19 is <5%, ~19% of hospitalized patients in France are transferred to the intensive care unit (ICU) [7]. The median length of l stay (LOS) for COVID-19 patients has been reported to be ≤8 days based on a Chinese study; however, larger studies may be needed to better understand the course of COVID-19 after ICU admission [6,11]. In addition, the LOS varied significantly between countries even before the pandemic [11]. In Saudi Arabia, the rate of hospitalization among all confirmed COVID-19 cases during March 2020 was 71.6% according to Alsofayan and colleagues, but the mortality rate was as low as 0.65% [12]. This high reported rate of hospitalization among COVID-19 cases may exacerbate the cost burden of viral respiratory infections in a country that was deeply affected by the Middle East Respiratory Syndrome (MERS) in 2012, and resulted in a huge financial burden with an estimated direct medical cost per patient of SAR 48,551.36 (United States dollars (USD) 12,947.03) [13]. 

In light of the high rate of hospitalization among COVID-19 patients in Saudi Arabia, there is a need to identify different sociodemographic (e.g., age, sex) and medical (e.g., mechanical ventilator (MMV) use, ICU admission) status that might increase mortality risk. Moreover, the cost of hospitalization should be estimated. Providing government officials and clinicians with clear guidance on the risk factors, mortality rate, and how to prioritize screening, testing, isolation or quarantining of COVID-19 cases is imperative to manage this pandemic effectively and efficiently. 

Here, we investigated the survival of hospitalized COVID-19 patients in Saudi Arabia across age groups, sex, nationality, MV use, and ICU admission. Furthermore, the average cost of hospitalization due to COVID-19 per patient was estimated. 

## 2. Methods

### 2.1. Ethical Approval of the Study Protocol

This study protocol was approved by the Ethics Review Board Committee of the Central Ministry of Health (20–75M) in Riyadh, Saudi Arabia.

### 2.2. Data Source

The data of this study were retrieved from the Health Electronic Surveillance Network (HESN) database of the Saudi Ministry of Health (MoH) for COVID-19 patients. All symptomatic patients with confirmed COVID-19 after being tested in outpatient settings and confirmed in inpatient settings upon admission in Saudi hospitals from 1 March to 29 May 2020 were included. The retrieved variables were age, sex, nationality (Saudi vs. non-Saudi), city, hospital, date of hospital admission, date of discharge from hospital, MV use, inpatient environment (ICU vs. General Medical Ward (GMW)), and final status (discharge vs. death). Data on comorbidities were missing for most cases. No re-admissions for the COVID-19 patients were encountered in the retrieved data. All consecutive patients were assumed to receive standardized treatment protocols for COVID-19 as posted on the MoH website, and these protocols were (and are still being) updated on a regular basis. 

The cost of hospitalization was estimated using the micro-costing method as stated in the protocols for COVID-19 management set by the MoH. The cost of hospitalization was based on the cost of: all medications (e.g., antivirals, antibiotics, anticoagulants, hydroxychloroquine); personal protective equipment (e.g., N95 masks, gowns, protective eyewear); oxygen; MVs; isolation-room fees (ICU vs. GMW); fees of physicians and other medical staff; laboratory and diagnostic tests (e.g., polymerase chain reaction, complete blood count, liver/cardiac enzymes, swabs, cultures, radiographs and computed tomography of the chest). Data on inpatient costs were retrieved from the MoH Cost Center. The cost is presented in Saudi riyals (SAR). 

### 2.3. Inclusion/Exclusion Criteria

This was a retrospective cohort study upon which COVID-19 patients were followed up retrospectively between 1 March and 29 May 2020 from the date of hospital admission to discharge from hospital with final status which was either death or discharged alive (censored). Those without any update on their status were excluded.

### 2.4. Statistical Analyses

Kaplan–Meier (KM) survival analyses were created to examine the survival probability overall as well as across age groups. Moreover, the survival probability was estimated across MV use and sex, nationality (Saudi vs. non-Saudi), and inpatient environment (ICU vs. GMW). Comparisons of different strata were adjusted using Tukey’s method. The hazard ratio (HR) for death was generated using multiple Cox proportional-hazards regression that included the variables of: MV use (no vs. yes), age, sex (female vs. male), and inpatient environment (ICU vs. GMW). Significance was considered at α < 0.05, and the 95%CI is shown for different strata in all KM survival curves and reported for all HRs. Statistical analyses were conducted using SAS^®^ v9.4 (SAS, Cary, NC, USA). 

## 3. Results

**Figure 1:**
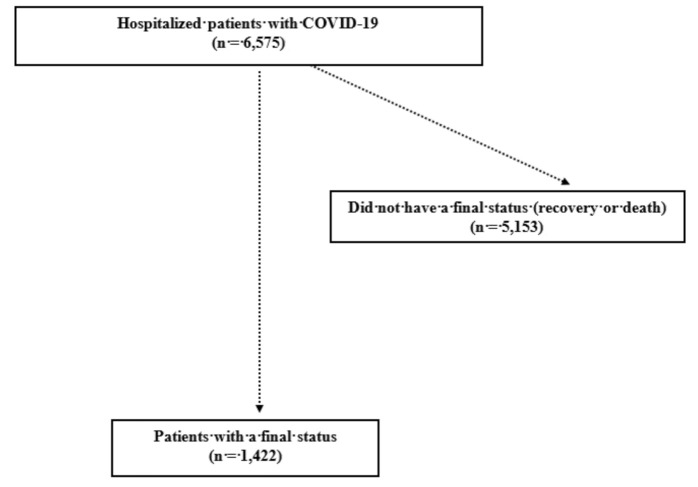
Patient inclusion flowchart.

The number of patients hospitalized due to COVID-19 between 1 March 2020 and 29 May 2020 was 6575. However, 5153 patients were not listed as having a final status (discharged alive or death) in the HESN database as of 29 May 2020. Therefore, only 1422 patients with final status (discharged alive or death) were eligible to be included in our analyses (Figure 1).

The majority of the patients were male (77.71%), and between 25 and 54 years of age (67.65%). Most patients were non-Saudi (68.78%), and from Medina (57.95%). Only 15% of patients were admitted to the ICU, and MV use was indicated in 13% (Table 1). About 16% of patients (263 patients) died in hospital. The median LOS was 7.93 days, with a maximum LOS of 43 days. The overall mean survival time from admission to final status (discharged alive or death) for the study cohort was 21 days with differences across different variables (Table 2). Older COVID-19 patients had a significantly shorter mean duration of survival compared with their younger counterparts (*p* < 0.0001). Patients on MVs had a significantly shorter mean duration of survival compared with those not on MVs (8.87 vs. 18.93 days, *p* < 0.0001). Likewise, those admitted in ICUs had a significantly shorter mean duration of survival compared with those admitted to other inpatient environments (11.13 vs. 18.89 days, *p* < 0.0001).

**Figure 2:**
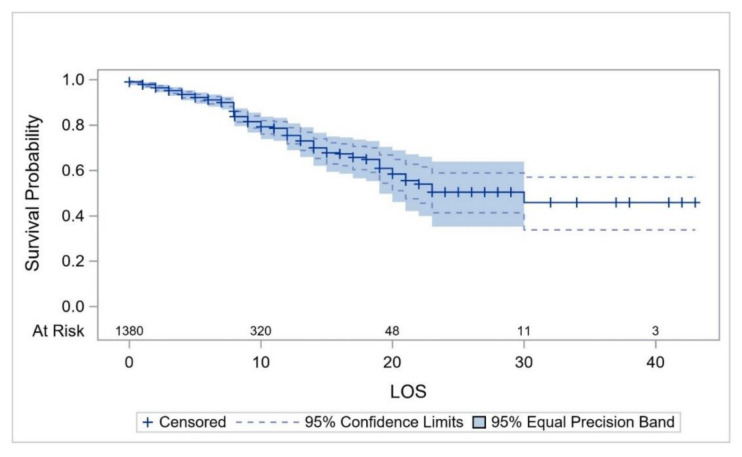
The overall survival probability of hospitalized COVID-19 patients over their LOS. Abbreviations: LOS = length of stay.

**Figure 3:**
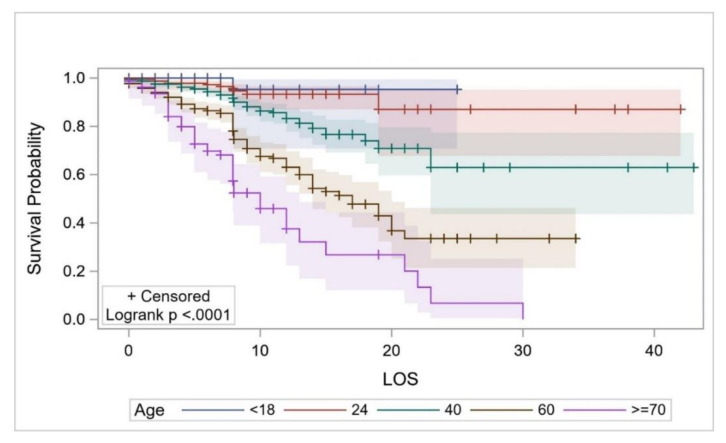
The survival probability of COVID-19 patients across different age groups over their LOS. Abbreviations: LOS = length of stay.

**Figure 4:**
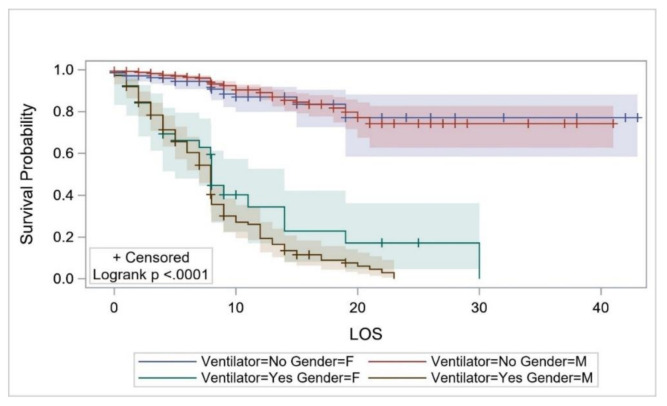
The survival probability of COVID-19 patients across sex and mechanical-ventilator use over their LOS. Abbreviations: LOS = length of stay, M = Male, F = Female.

**Figure 5:**
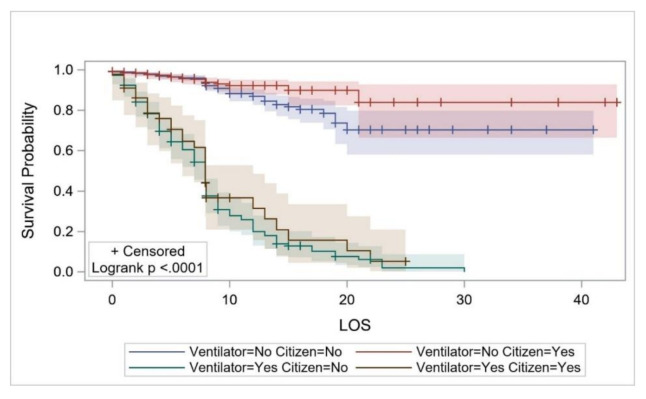
The survival probability of COVID-19 patients across citizenship status and mechanical-ventilator use over their LOS. Abbreviations: LOS = length of stay.

**Figure 6:**
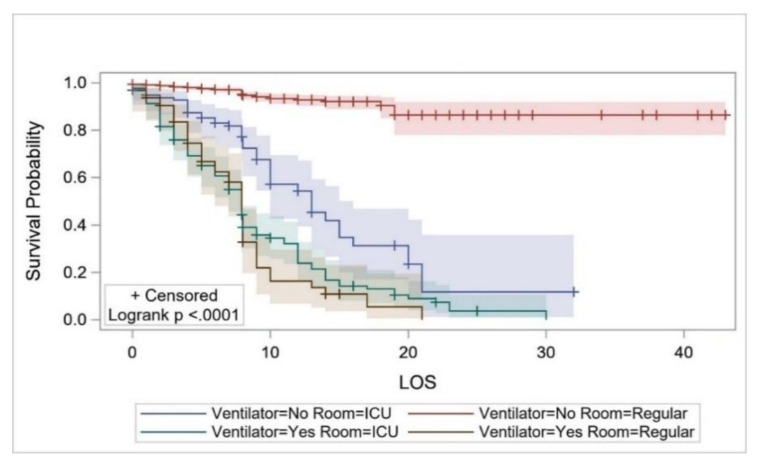
The survival probability of COVID-19 patients across inpatient environment (GMW vs. ICU) and mechanical-ventilator use over their LOS. Abbreviations: LOS = length of stay, GMW = general medical ward, ICU = intensive care unit.

**Figure 7:**
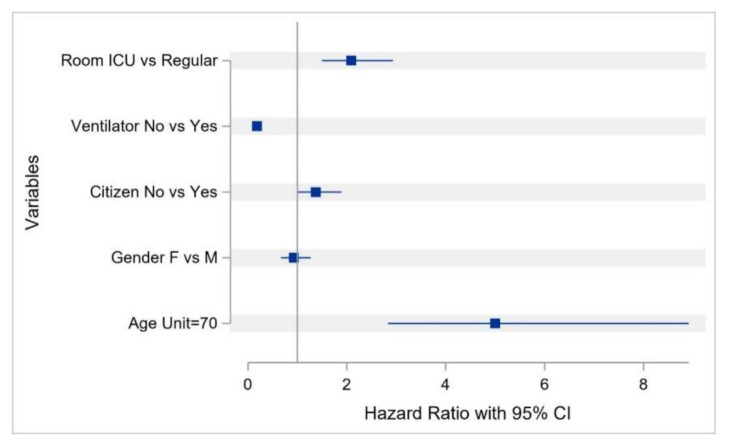
The adjusted hazard ratios for death.

The survival probability (which was estimated using KM curves in all cases) of the overall study cohort from hospital admission up to the second day of hospitalization was estimated to be 0.978 (95%CI: 0.969–0.985) but decreased to 0.699 (0.652–0.741) on day 14, and to 0.459 (0.339–0.571) on day 30 (Figure 2). The survival probability for COVID-19 patients ≤18 years of age for ≥8 days was estimated to be 0.952 (95%CI: 0.707–0.993). The survival probability of patients aged ≥70 years for 8 days and up to 10 days was 0.524 (95%CI: 0.389–0.642), and 0.067 (0.005–0.251) for 23 days (Figure 3). The survival probability of male and female patients not on MVs for 19 days was 0.797 (95%CI: 0.716–0.857) and 0.771 (0.584–0.881), respectively. The survival probability of male and female patients who were on MVs for 19 days was 0.076 (95%CI: 0.034–0.141) and 0.172 (0.047–0.362), respectively (Figure 4). The survival probability for Saudi citizens and non-Saudi patients who were not on MVs for 20 days was 0.838 (95%CI: 0.663–0.927) and 0.703 (0.580–0.796), respectively. The survival probability for Saudi and non-Saudi patients who were on MVs was 0.105 (95%CI: 0.019–0.274) and 0.062 (0.023–0.127), respectively (Figure 5). The survival probability for 20 days of patients not on MVs but admitted to the ICU was 0.235 (95%CI: 0.088–0.421), and 0.864 (0.779–0.917) for those on the GMW. For patients who were on MVs, the survival probability for 20 days was 0.089 (95%CI: 0.040–0.163) for those admitted to the ICU, and 0.054 (0.005–0.193) for those admitted to the GMW (Figure 6). For each 1-year increase in age, the death risk increased by an estimated 2.3% (HR = 1.023, *p* < 0.0001). The risk of death among patients on MVs was five-times higher compared with their counterparts who were not on MVs (HR = 5.15, *p* < 0.0001). The death risk for non-Saudi patients was 36% higher than that of their Saudi counterparts (HR = 1.36, *p* = 0.049). Furthermore, the death risk for patients admitted to the ICU was more than twice that of their counterparts admitted to the GMW (HR = 2.08, *p* < 0.0001). Being female was not associated with a lower risk of death (HR = 0.944, *p* = 0.725). The adjusted HRs with their 95%CIs are shown in Table 3 and Figure 7.

**Figure 8:**
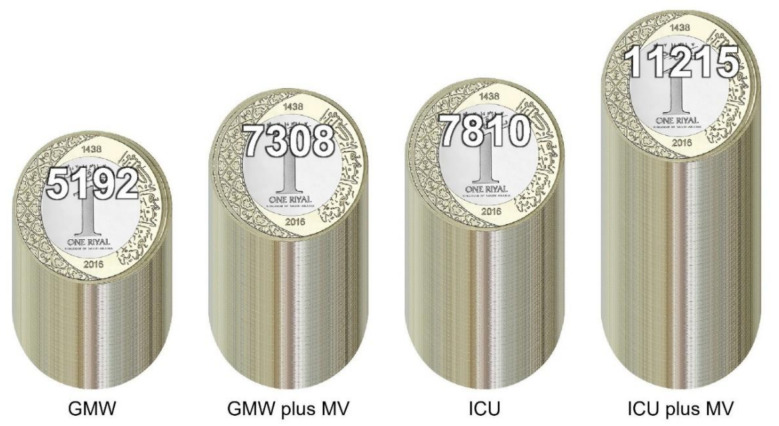
The mean cost of COVID-19 hospitalization per patient per day in Saudi Riyals (SAR). Abbreviations: GMW = general medical ward, GMW plus MV = general medical ward and on invasive mechanical ventilator, ICU = intensive care unit, ICU plus MV = intensive care unit and on invasive mechanical ventilator.

The mean hospitalization cost per patient per day for those admitted to the GMW was SAR 5192.15 ± 622.64 (USD 1384.57 ± 166.04) and SAR 7307.62 ± 2127.45 (USD 1948.69 ± 567.32) for those not on MVs and those on MVs, respectively. The mean cost per patient (in SAR) for those admitted to the GMW and ICU was 42,704.49 ± 29,811.25 (USD 11,387.86 ± 7949.66) and 79,418.30 ± 55,647.69 (USD 21,178.21 ± 14,839.38), respectively. The mean hospitalization cost per patient per day for patients admitted to the ICU and not on MVs was SAR 7809.94±1293.91 (USD 2082.65 ± 345.04), and SAR 11,215.36 ± 2047.43 (USD 2990.76 ± 545.98) for those on MVs (Figure 8). The mean hospitalization cost for patients admitted to the ICU and not on MVs was lower than that for their counterparts on MVs (SAR 73,289.57 vs. SAR 84,174.19). The mean hospitalization cost (in SAR) for patients admitted to the GMW and not on MVs was slightly higher than that of their counterparts on MVs (42,712.59 vs. 42,558.34) (Table 4). Overall, the mean hospitalization cost per patient (in SAR) was 48,436.18 ± 37,539.05 and the median was 38,436.71 (IQR, 29,787.80–52,194.30).

## 4. Discussion

The COVID-19 pandemic has had a detrimental effect on global healthcare systems, and affected every aspect of human and economic life [14]. As of 22 June 2020, the number of COVID-19 cases in Saudi Arabia has exceeded 161,000, with an estimated case fatality rate (CFR) of 0.81% [15]. The reported CFR is far below that of France, Belgium, Spain, Italy, and the UK, which have reported a CFR between 11.5% (Spain) to 16% (Belgium) [1,5,16]. However, the CFR among hospitalized COVID-19 patients is far higher than the population-level CFR. Our study (which is the first to report the survival probability across age groups, sex, nationality, MV use, and ICU admission among a sample of hospitalized COVID-19 patients in Saudi Arabia) revealed the CFR to be 16.6%. This CFR is far below the reported CFR among hospitalized patients in the UK (33%) [17] (Docherty et al., 2020), Italy (27%) [18], and the USA (21%) [19]. In addition, the percentage of hospitalized patients treated in the ICU or who received invasive ventilation was similar to the one reported in the USA [19]. 

The overall 30-day mortality among our cohort was 16.6%, but the 14-day mortality (15.81%) represented >91% of deaths. This finding suggests that the first 14 days of hospitalization are critical for COVID-19 patients, which has also been reported among a sample of hospitalized Italian patients with COVID-19 [18]. The overall 14-day and 30-day survival probability (using KM curves) was 0.699 and 0.459, respectively. This observation is consistent with a study conducted in Sichuan Province in China, which found that the LOS was associated with higher risk of death [20]. Older adults were at a significantly higher risk of death compared with those in other age groups, a finding that is in accordance with the work of other scholars [21,22]. This higher risk of mortality among ICU patients aligns with the findings of research studies among hospitalized COVID-19 patients [23]. Although most hospitalized patients were male, the risk of mortality was not higher among male patients in comparison with their female counterparts. This finding contradicts the observations of other scholars who showed a higher risk of mortality among hospitalized male patients with COVID-19 [24,25]. Patients on MVs had a more than five-times higher risk of death compared with their counterparts not on MVs, a finding that is similar to data from Auld and colleagues [26]. Pareek and collaborators reported that ethnicity may have a role in the survival of COVID-19 patients [27]. We found that hospitalized non-Saudi patients were at a slightly higher risk of mortality. This could be attributable to the fact that many non-Saudi patients who were hospitalized for COVID-19 did not have legal residence status, and lack health insurance coverage prior to the COVID-19 pandemic. However, this could change if other diseases (e.g., diabetes mellitus, asthma, hypertension, cardiovascular diseases, or chronic renal failure) were controlled for in the analysis.

A major concern about the COVID-19 pandemic is the high cost burden to healthcare systems. We calculated the direct medical cost associated with treatment of COVID-19 patients in Saudi Arabia. The cost of COVID-19 treatment was calculated based on MoH treatment protocols and accounted for all health resources used to deliver care to COVID-19 patients. Our cost data highlighted differences in resource utilization between patients presenting with moderate-to-severe symptoms versus critical cases who required ICU admission. Our cost analyses illustrated that the mean direct medical cost of patients with moderate-to-severe COVID-19 symptoms admitted to the GMW was SAR 5303.21per patient per day, which was much lower compared with the mean cost per patient per day for patients admitted to the ICU (e.g., SAR 9727.41). However, the difference in the mean cost per patient per day between patients who needed MVs and those who did not need them was SAR 2244.42 and SAR 3405.42 among patients admitted in GMW and ICU, respectively.

The total direct medical cost per patient was calculated based on the level of care and LOS. The total direct medical cost per patient for those with moderate-to-severe symptoms admitted to the GMW was SAR 42,704.49. However, there was an approximate twofold increase in the cost for ICU patients (e.g., SAR 79,418.30). Interestingly, the total cost for patients on MVs was slightly lower in comparison with their counterparts admitted to the GMW but who were not on MVs. This finding was largely attributable to a significantly shorter duration of survival and higher rate of mortality among patients on MVs, which translated to a shorter LOS and, eventually, lower total cost per patient. However, this finding is not consistent with data from a study by Rae and colleagues, who reported that patients on MVs often required a longer hospital stay with a higher cost of healthcare-resource utilization [28].

There is a dearth of data about the direct medical cost of COVID-19 in the Middle East. Very few scholars have assessed the financial impact on healthcare systems worldwide. The mean direct medical cost per patient (in USD) has been reported to be 2395 in China [29], 10,000 in Canada [30] and 4633.43 in India [31]. A study published recently in the USA reported the mean cost of treatment of patients with mild COVID-19 who were not hospitalized ranged from USD 32 for consultation over the telephone to USD 96 for a clinic visit [32]. Those data are in accordance with our observations because mild cases are often not hospitalized and used medications mainly for relief from fever or pain only. Moreover, that USA study estimated the median direct medical cost of caring for patients with moderate COVID-19 symptoms who did not require hospitalization but had to be seen at Emergency Department was USD 3045, and was USD 14,366 for those with more severe symptoms that necessitated hospitalization. Based on those estimates, the total direct medical cost in the USA has been projected to range from USD 163.4 billion to USD 654.0 billion [32]. In Sweden, the total direct medical cost has been projected to reach USD 2 billion [33]. The mean direct medical cost per patient we estimated was SAR 48,436.18 (USD 12,916.31), which was not significantly different from the one reported for the management of a MERS-CoV patient in Saudi Arabia (USD 12,947.03) [13]. However, the total direct medical cost of COVID-19 far exceeds the one reported for MERS-CoV due to the high number of COVID-19 infections that are being reported on a daily basis. These variations in cost estimates across countries highlight the challenges in estimating and comparing the direct medical costs globally given the vast differences in the cost of treatment protocols, personnel cost, and utilization rates of healthcare resources and their prices between countries.

Our study had four main limitations. First, we did not include all hospitalized patients in Saudi Arabia, which limits the generalizability of our findings. Second, variables such as comorbidities (e.g., diabetes mellitus, asthma, cancer, hypertension, chronic kidney disease), smoking status, and occupation were not investigated, which may have changed our findings if they had been controlled for in our analyses. Additionally, the study did not control for the changes in the treatment protocols and their potential impact on mortality rates. Third, this study was conducted from the perspective of healthcare payers, and did not take into consideration other important costs, such as productivity losses and “lockdown” costs. Therefore, the economic impact of COVID-19 would have been much greater. Fourth, the outcomes for patients who were discharged alive (censored) cannot be ascertained as some discharged patients may have died or readmitted afterwards.

Future research should examine the: (i) survival probability for hospitalized COVID-19 patients controlling for comorbidities and other potential confounders; (ii) cost of COVID-19 on other important sectors of the economy; (iii) total direct medical costs of COVID-19 to the Saudi Arabia healthcare system.

## Figures and Tables

**Table 1:** Baseline characteristics of study cohort (*n* = 1422).

Characteristic	N (%)
Sex	
Male	1105 (77.71)
Female	317 (22.29)
Age group (years)	
0–4	19 (1.34)
5–14	24 (1.69)
15–24	77 (5.41)
25–34	351 (24.68)
35–44	317 (22.29)
45–54	294 (20.68)
55–64	191 (13.43)
65–74	98 (6.89)
75–84	36 (2.53)
≥85	15 (1.05)
Nationality	
Saudi	444 (31.22)
Non-Saudi	978 (68.78)
City	
Al Ahsa	82 (5.77)
AlBahah	39 (2.74)
Sakaka	4 (0.28)
Khamis Mushait	11 (0.77)
Bisha	5 (0.35)
Hafar Al Batin	2 (0.14)
Hail	38 (2.67)
Jizan	16 (1.13)
Makkah	205 (14.42)
Medina	824 (57.95)
Najran	37 (2.60)
Arar	2 (0.14)
Buraydah	52 (3.66)
Al Qunfudhah	5 (0.35)
Riyadh	4 (0.28)
Tabouk	39 (2.74)
Taif	57 (4.01)
Inpatient environment	
GMW	1200 (84.39)
ICU	222 (15.61)
Use of mechanical ventilator	
Yes	188 (13.22)
No	1234 (86.78)

Abbreviations: GMW= general medical ward, ICU = intensive care unit.

**Table 2:** Mean survival time in days from admission to final status (discharged alive or death) and patients’ characteristics.

Characteristic	Mean Survival ± SE	P
Overall	21.44 ± 0.55	—
Sex		
Male	17.78 ± 0.34	0.597
Female	22.81 ± 1.08	
Age (years)		
30 to <50	18.12 ± 0.29	<0.0001 *
50 to <70	14.63 ± 0.47	
≥70	12.01 ± 1.34	
Use of mechanical ventilator		
Yes	8.87 ± 0.55	<0.0001 *
No	18.93 ± 0.21	
Inpatient environment		
ICU	11.13 ± 0.66	<0.0001 *
GMW	18.893 ± 0.21	

Abbreviations: ICU = intensive care unit, GMW = general medical ward. * *p* < 0.05.

**Table 3:** Multiple Cox-proportional hazard model for mortality among COVID-19 patients.

Variable	HR (95%CI)	*P*
Age	1.023 (1.01–1.03)	<0.0001 *
Sex (female vs. male)	0.944 (0.68–1.30)	0.725
Use of mechanical ventilator (no vs. yes)	0.194 (0.137–0.273)	<0.0001 *
Nationality (non-Saudi vs. Saudi)	1.36 (1.001–1.865)	0.049 *
Inpatient environment (ICU vs. GMW)	2.088 (1.495–2.916)	<0.0001 *

HR = hazard ratio, ICU = intensive care unit, GMW = general medical ward, CI = confidence interval. * *p* < 0.05.

**Table 4:** Estimated direct medical costs in Saudi Riyals (SAR) based on the length of stay across the inpatient environment and mechanical-ventilator use.

Inpatient Setting	Mechanical-Ventilator Use	Number of Patients	Mean ± SD	Median (Q1-Q3)
ICU	No	97	73,289.57 ± 47,216.44	54,603.92 (54,603.92–81,140.48)
	Yes	125	84,174.19 ± 61,162.84	78,625.02 (44,080.16–88,030.36)
GMW	No	1137	42,712.59 ± 30,048.16	38,436.71 (25,306.50–43,231.70)
	Yes	63	42,558.34 ± 25,360.95	38,207.80 (28,445.20–47,970.40)

Abbreviations: ICU = intensive care unit, GMW = general medical ward.
