# Erratum: Khan, A.A., et al. Survival and Estimation of Direct Medical Costs of Hospitalized COVID-19 Patients in the Kingdom of Saudi Arabia (Short Title: COVID-19 Survival and Cost in Saudi Arabia). *Int. J. Environ. Res. Public Health* 2020, *17*, 7458

**DOI:** 10.3390/ijerph17249458

**Published:** 2020-12-17

**Authors:** Anas A. Khan, Yazed AlRuthia, Bander Balkhi, Sultan M. Alghadeer, Mohamad-Hani Temsah, Saqer M. Althunayyan, Yousef M. Alsofayan

**Affiliations:** 1 Department of Emergency Medicine, College of Medicine, King Saud University, Global Center for Mass Gatherings Medicine, Ministry of Health, Riyadh 11451, Saudi Arabia; anaskhan@KSU.EDU.SA; 2 Department of Clinical Pharmacy, College of Pharmacy, King Saud University, P.O. Box 2454, Riyadh 11451, Saudi Arabia; bbalkhi@KSU.EDU.SA (B.B.); salghadeer@ksu.edu.sa (S.M.A.); 3 Pharmacoeconomics Research Unit, College of Pharmacy, King Saud University, P.O. Box 2454, Riyadh 11451, Saudi Arabia; 4 College of Medicine, King Saud University, Riyadh 11451, Saudi Arabia; mtemsah@ksu.edu.sa; 5 Department of Pediatrics, King Saud University Medical City, King Saud University, Riyadh 11451, Saudi Arabia; 6 Department of Accident and Trauma, Prince Sultan Bin Abdulaziz College for Emergency Medical Services, King Saud University, Riyadh 11451, Saudi Arabia; salthunayyan@KSU.EDU.SA; 7 Global Center for Mass Gatherings Medicine, Ministry of Health, Riyadh 11451, Saudi Arabia; y-m-alsofayan@hotmail.com

1. There is an error in the title. The correct title of the article [1] is “Survival and Estimation of Direct Medical Costs of Hospitalized COVID-19 Patients in the Kingdom of Saudi Arabia”. We apologize for this error and state that the scientific conclusions are unaffected. The original article has been updated.
2. There is a typo mistake in the abstract’s conclusion where “is” which follows “represents” should be deleted so that the sentence can be grammatically correct, as highlighted in the following text: “Conclusion: The high hospitalization costs for COVID-19 patients represents is a significant public health challenge. Efficient allocation of healthcare resources cannot be emphasized enough.”

The authors would like to apologize for any inconvenience caused to the readers by these changes.
